# Differential Oxidative Stress Induced by Dengue Virus in Monocytes from Human Neonates, Adult and Elderly Individuals

**DOI:** 10.1371/journal.pone.0073221

**Published:** 2013-09-17

**Authors:** Nereida Valero, Jesús Mosquera, Germán Añez, Alegria Levy, Rafael Marcucci, Melchor Alvarez de Mon

**Affiliations:** 1 Instituto de Investigaciones Clínicas “Dr. Américo Negrette”, Facultad de Medicina, Universidad del Zulia, Maracaibo, Venezuela; 2 Catedra de Embriologia, Escuela de Bioanalisis, Facultad de Medicina, Universidad del Zulia, Maracaibo, Venezuela; 3 Department of Immune System Diseases and Oncology, University Hospital “Príncipe de Asturias”, Alcala University, Madrid, Spain; Department of Immunology, China

## Abstract

Changes in immune response during lifespan of man are well known. These changes involve decreased neonatal and elderly immune response. In addition, it has been shown a relationship between immune and oxidative mechanisms, suggesting that altered immune response could be associated to altered oxidative response. Increased expression of nitric oxide (NO) has been documented in dengue and in monocyte cultures infected with different types of dengue virus. However, there is no information about the age-dependent NO oxidative response in humans infected by dengue virus. In this study, monocyte cultures from neonatal, elderly and adult individuals (n = 10 each group) were infected with different dengue virus types (DENV- 1 to 4) and oxidative/antioxidative responses and apoptosis were measured at days 1 and 3 of culture. Increased production of NO, lipid peroxidation and enzymatic and nonenzymatic anti-oxidative responses in dengue infected monocyte cultures were observed. However, neonatal and elderly monocytes had lower values of studied parameters when compared to those in adult-derived cultures. Apoptosis was present in infected monocytes with higher values at day 3 of culture. This reduced oxidant/antioxidant response of neonatal and elderly monocytes could be relevant in the pathogenesis of dengue disease.

## Introduction

Monocytes/macrophages (Mo/MΦ) represent one of the important targets during dengue infection and are important in viral dissemination [1]–[3]. It has been shown that dengue virus (DENV) is capable of inducing oxidative stress in humans [4]–[6] suggesting that the interaction of DENV with Mo /MΦ could play a role in the pathogenesis of dengue. It has also been reported that the immune alterations can influence oxidative metabolism and vice versa [7]. In this regard, monocytes from neonates and elderly individuals have been shown to have immunosuppressive status against infections [8]–[15], suggesting a possible altered oxidative response. Nitric oxide (NO) plays an important role in inflammation and in the regulation of immune responses [16], [17]. This nitrogen reactive species are greatly produced by Mo/MΦ during inflammatory processes [7]. Since, an altered immune response has been documented in neonatal and elderly monocytes, we hypothesized that neonatal and elderly monocytes probably have an altered oxidative response to dengue infection. Therefore, the aim of this study was to analyze the oxidant (nitric oxide) and antioxidant (catalase, superoxide dismutase and reduced glutathione) responses of monocyte from neonates, young adults and elderly subjects during an *in vitro* dengue virus infection. During this study both elderly and neonatal monocytes had lower oxidant/antioxidant responses to dengue virus infection. These findings are probably important in the pathogenesis of dengue disease in individuals from those age groups.

## Materials and Methods

### Preparation of virus stock and virus titration

DENV prototype laboratory strains; DENV-1 (Hawaii), DENV-2 (New Guinea C), DENV-3 (H-87) and DENV-4 (H-241) were propagated in mosquito C6/36HT cells that were cultured in Eagle's MEM medium containing 10% FBS prior to viral monocyte infection. The virus culture medium was harvested after 5 days of incubation and after removal of cell debris by centrifugation, the supernatant containing virus was aliquoted and stored at −70°C until used. Virus was titrated by plaque formation assay on Vero cells [18]. Cells were planted at 1×10^6^ cells/well in 24-well plates and subsequently, serial dilutions of virus were added and incubated at 37°C for 7 days. Afterwards, the plaques were visualized by staining with 1% crystal violet solution. Virus concentration is given as plaque-forming units (PFU)/ml. Virus stock was free of endotoxin as determined by limulus amebocyte lysate assay (Charles River, MA, USA).

### Monocyte cultures

Monocytes were isolated from heparinized peripheral blood obtained from male human healthy neonates (umbilical cord), young adults (35–45 years old) and elderly (65–70 years old) subjects (N = 10 each group). All individuals were tested for circulating NS1 protein and anti- DENV antibodies (Dengue NS1 Ag + Ab combo, Standard Diagnostic, Inc. Bioline, Korea). Subjects positive to DENV NS1 protein or anti-DENV antibodies were excluded from this study. Mononuclear cells were obtained by density centrifugation over 1.077 Histopaque (Sigma Chemical Co, St. Louis, MO). Individuals or parents were informed about the study procedures and their written consents were obtained before enrollment in the investigation. In this context, individuals or their relatives were informed of the scope of the study, samples to take and obtained results. Written consents were approved by the Bioethical Committee of Medical School (Universidad del Zulia, Maracaibo, Venezuela).

Total mononuclear leukocytes recovered from the interface were washed and suspended in RPMI 1640, 10% fetal bovine serum and penicillin/streptomycin. Afterwards, 300 μl/well of a cellular suspension (4×10^6^ cells/ml) were layered on 24-well plastic tissue culture plates (Nunc, Roskilde, Denmark) and incubated for 3 hours at 37°C and 5% CO_2_. Non adherents cells were washed out with warm medium and adhered cells (approx. 3×10^5^ cells) were used for experiments. The monolayers of adherent cells were reacted with an FITC conjugated anti- human CD14, monoclonal antibody (Sigma Chemical Co., St. Louis, MO, USA) to determine monocyte percentage, using a microscopy with epifluorescence system (Zeiss, Germany).

### Infection of monocyte cultures

Monocytes from each subject were infected with the different DENV types at a multiplicity of infection of 1 (MOI: 1) and incubated for 1 and 3 days at 37°C and 5% CO_2_. Controls represent monocytes cultured with supplemented medium without virus. In addition, monocyte cultures were incubated with LPS (50 ng/ml) (Sigma-Aldrich Company, St. Louis MO, USA) for the same period of time. Doses of MOI: 1 and LPS (50 ng/ml) were chosen, since they were capable of inducing high production of proinflammatory cytokines under the same culture conditions (unpublished data).

### Determination of nitrite/nitrate production

Total nitrite concentration in monocyte homogenates was used as an indicator of nitric oxide (NO) synthesis. Nitrates in samples were reduced to nitrites by incubating with nitrate reductase. Nitrite concentration was measured using a commercial kit following the manufacturer's indications (Nitric Oxide Quantichrom, Bioassay Systems, Hayward, USA). Optical density was measured at 550 nm and results expressed as μM/mg of cellular protein.

### Determination of thiobarbituric reactive substances (TBARS)

Monocyte malondialdehyde (MDA) content was assessed by the thiobarbituric acid assay (NWLSS, Malondialdehyde Assay. Vancouver, WA). Absorbance was measured at 532 nm. As external standard the MDA bisdimethyl acetal (Sigma – Aldrich, St. Louis, MO, USA) was used and results were expressed as nmol per mg of cellular protein.

### Determination of enzyme activities and reduced glutathione (GSH)

Treated monocyte cultures and controls were homogenized and catalase activity was determined using a commercial kit (Cayman Chemical Company, Michigan, USA). Results are expressed as nmol/min/mg of cellular protein. Superoxide dismutase (SOD) activity was determined using a commercial kit (Cayman Chemical Company, Michigan, USA) and results were expressed as as U /mg of cellular protein. Content of GSH was also determined in monocyte homogenates using a commercial kit following the manufacturer's indications (Cayman Chemical Company, Michigan, USA). Results were expressed as μM/mg of cellular protein. Total protein content was measured in the monocyte homogenates by the method of Bradford (Bio Rad, USA).

### Determination of apoptosis

Controls, LPS and infected monocyte cultures were fixed with 1% paraformaldehyde in PBS for 10 min at room temperature and permeabilized with acetic acid: ethanol. The percentages of apoptotic cells were assessed by TUNEL reaction using the *In situ* Apop Tag kit (Chemicon International, USA & Canada) according to the manufacturer's instructions.

### Statistical analysis

Data were expressed as mean ± standard deviation. Differences between groups were determined by ANOVA followed by Bonferroni posttest. Significance was assumed to be at two tailed p<0.05.

## Results

**Figure 1:**
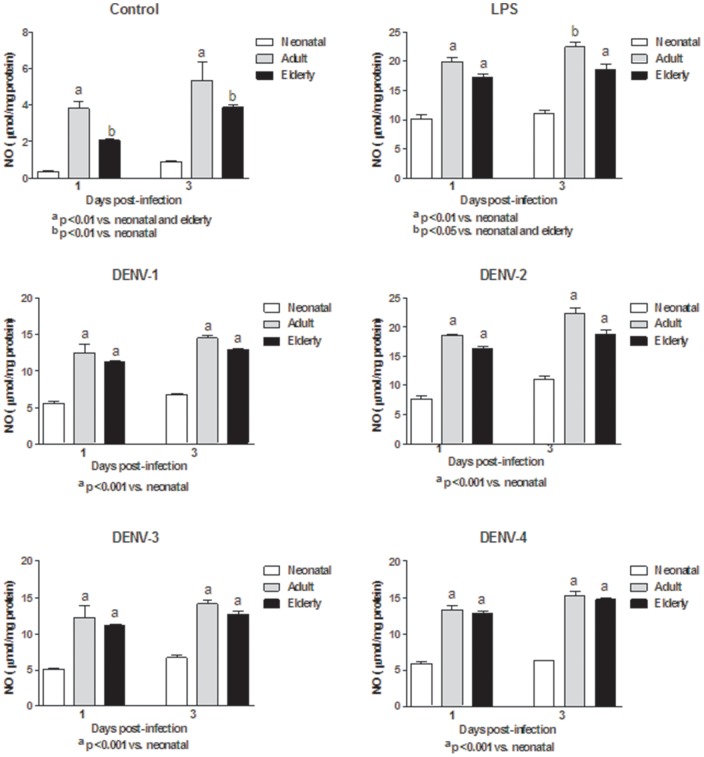
Induction of nitric oxide (NO) by different DENV types and LPS according to monocyte source. Decreased NO values in neonatal and elderly monocyte cultures infected with all DENV types compared to adult monocyte cultures were observed at days 1 and 3. These responses were similar to those observed with LPS stimulation.

**Figure 2:**
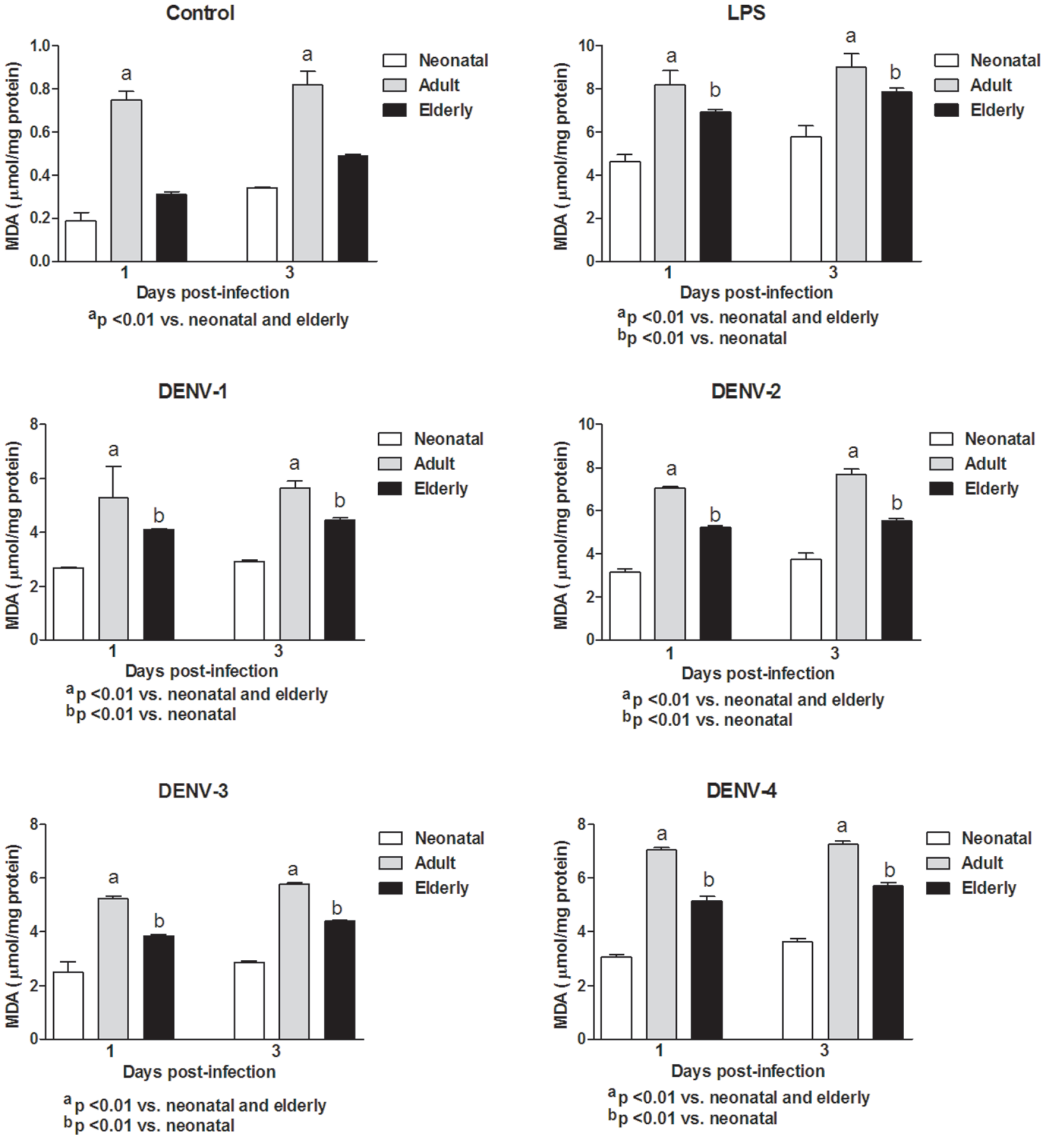
Lipid peroxidation (MDA) induced by different DENV types and LPS according to monocyte source. Lower values of MDA in neonatal and elderly monocyte cultures than those in adult cultures were observed at days 1 and 3. These responses were similar to those observed with LPS stimulation.

**Figure 3:**
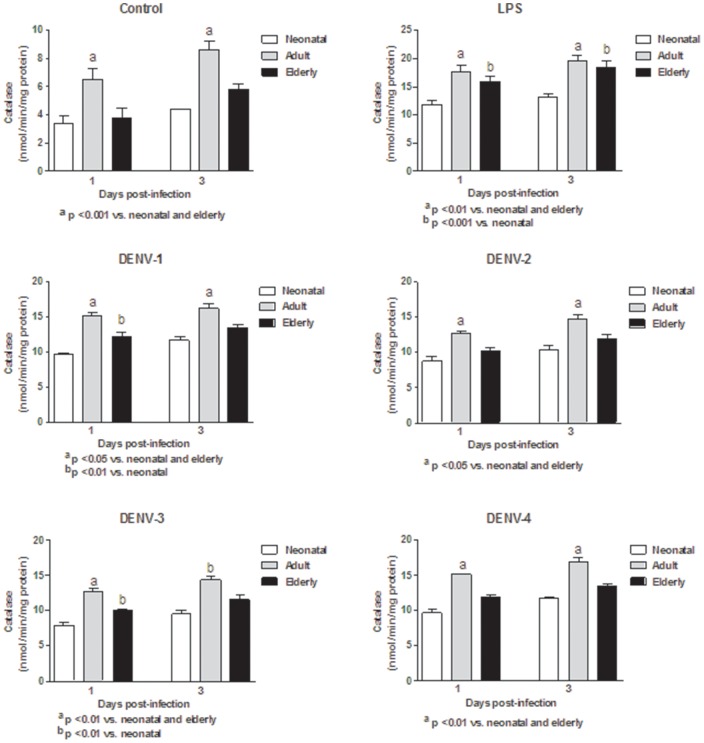
Induction of catalase activity by different DENV types and LPS according to monocyte source. Decreased values in neonatal and elderly monocyte cultures infected with all DENV types compared to adult monocyte cultures were observed at days 1 and 3. These responses were similar to those observed with LPS stimulation.

**Figure 4:**
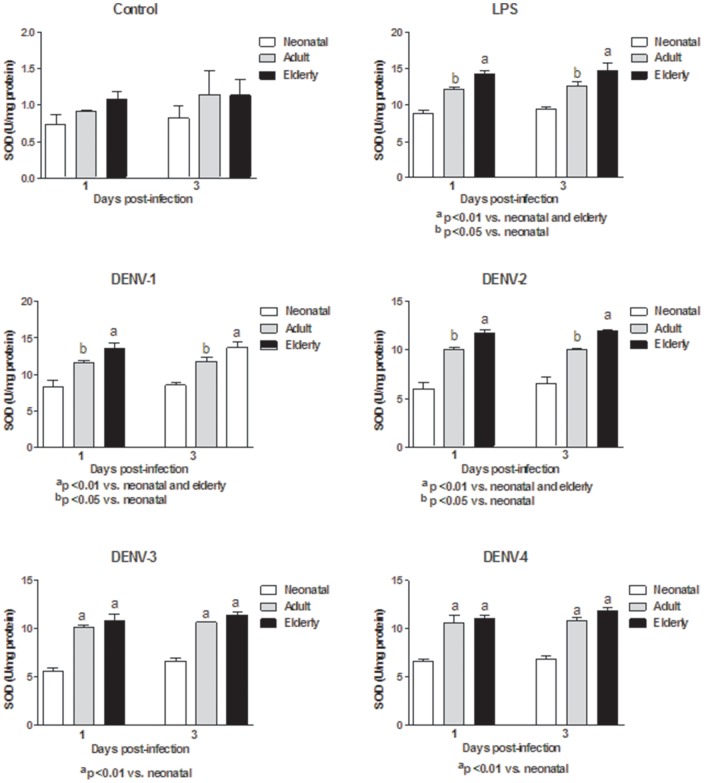
Induction of superoxide dismutase (SOD) activity by different DENV types and LPS according to monocyte source. Decreased activity in neonatal monocyte cultures infected with all DENV types compared to adult and elderly monocyte cultures were observed at days 1 and 3.

**Figure 5:**
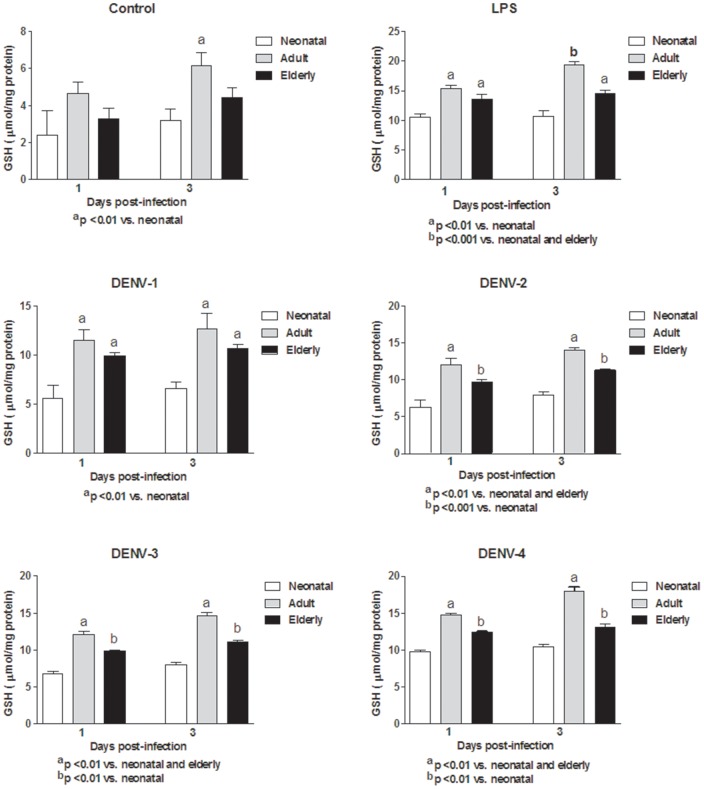
Reduced glutathione (GSH) content in dengue virus or LPS treated monocyte cultures according to monocyte source. Decreased values in neonatal and elderly monocyte cultures infected with all DENV types compared to adult monocyte cultures were observed at days 1 and 3. These responses were similar to those observed with LPS stimulation.

**Figure 6:**
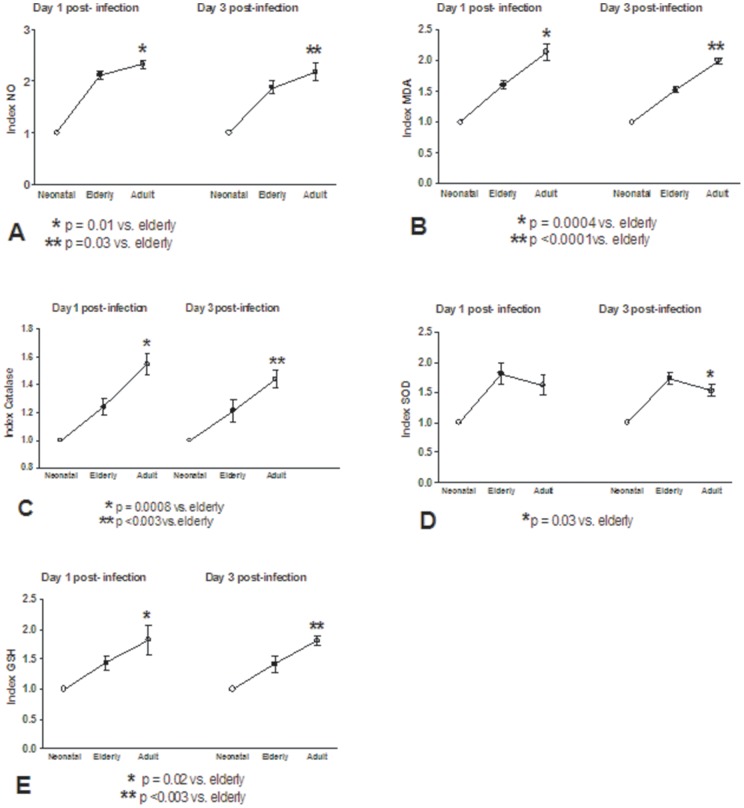
Index elderly or adult values/neonatal values. In general, values from neonatal cultures were lower than those observed in elderly and adult monocyte cultures in dengue infections (A, B, C, E) with the highest values in adult cultures; except for SOD activity with higher values in elderly cultures (D). These data represent the mean ± SD of the values from all DENV types.

**Figure 7:**
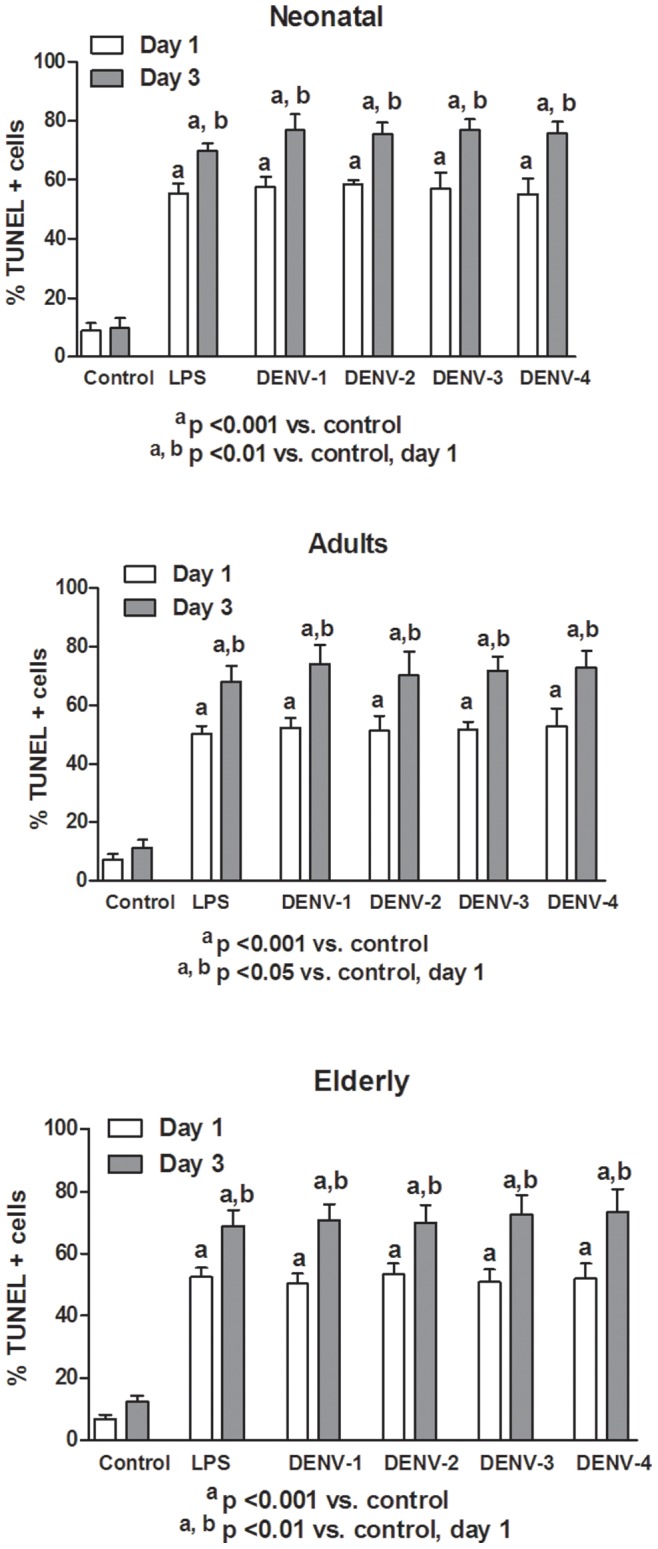
Induction of apoptosis (TUNEL) by different DENV types and LPS according to monocyte source. Increased apoptosis in LPS or infected-monocyte cultures, with higher values at day 3 were observed.

In this study, monocytes (purity >95%) were co-cultured with different DENV types or with LPS. In order to determine the response capacity of monocytes according to the age of the donor, monocytes from neonates, young adults and elderly individuals were tested for the oxidant/antioxidant response during infection by dengue virus. In general, lower values in NO and MDA productions (Figures 1 and 2; Tables S1 and S2), catalase and SOD activities (Figures 3 and 4; Tables S3 and S4) and GSH content (Figure 5; Table S5) were found in neonatal-monocytes, following by elderly-monocytes and the highest values were observed in adult-monocytes (Figure 6); except increased activity of SOD observed in elderly monocytes (Figure 6D). These findings were observed at days 1 and 3 post-infection and the pattern of monocyte response induced by dengue virus was similar to that observed in LPS-treated cultures (data no shown). Since oxidative stress is related to apoptosis, TUNEL assay was used to determine the degree of apoptosis in the different monocyte cultures. Apoptosis was increased in LPS and dengue virus infected-monocyte cultures, regardless of the monocyte source with higher values observed at day 3 (Figure 7, Table S6). To analyze the potential of different DENV types on the oxidative stress induction, monocytes cultures were infected with laboratory strains of DENV-1 to 4. Increased production of NO, MDA, catalase and SOD activities and GSH content were induced by the different viral serotypes and LPS in monocytes from neonatal, adult and elderly individuals. (as described in Figures S1-5). DENV-2 induced the highest production of NO accompanied with high production of MDA (Figures S1 and S2). Anti-oxidant response was also observed in all DENV types. The highest values of catalase activity were observed for DENV-1 and DENV-4 (Figure S3) and SOD activity in DENV-1 (Figure S4). The highest content of GSH was observed in monocytes infected by DENV-4 (Figure S5). The grade of oxidant and anti-oxidant responses was influenced by monocyte source.

## Discussion

NO and reactive oxygen species have modulating effects on inflammation and in the regulation of immune responses [16], [17]. During inflammatory reactions, Mo/MΦ are capable of producing increased amount of oxidants including NO [7]. Sustained inflammation resulting in inflammatory sequelae in neonates, is well known [9], suggesting altered monocyte function. In this study, we assessed the potential contribution of monocytes from neonates, elderly and young adult subjects for the production of NO and enzymatic and nonenzimatic antioxidants after infection with DENV. The neonatal monocyte response was lower than those observed in elderly and young adult monocytes, suggesting impaired response to dengue virus. There is little information about oxidative response by neonatal monocytes. However, it has been shown a relationship between immune and oxidative mechanisms [7], suggesting that altered immune response could be associated to altered oxidative response. Neonates are born with quantitative and qualitative defects in both adaptive and innate immune responses [10], [11]. In this regard, lymphocyte subset percentages in cord blood from neonates and cytokine responses to bacterial antigens were observed to be diminished when compared to peripheral blood from adults [19]. Plasma cytokine concentrations and cytokine production by neonatal monocytes after lipopolysaccharide stimulation *in vitro*, have been found decreased compared to adult plasma and monocytes [8]. In addition, blocking cytokine condition has been reported in neonates. Plasma interleukin-1 receptor antagonist was significantly higher in neonates than in plasma from adults [20]. The reduced response of neonatal monocytes to DENV could also be related to the increment of distinct inhibitory receptors on neonatal peripheral blood immune cells that could play a role in regulation of the neonatal immune system [10]. However, previous report has shown that the cord blood mononuclear phagocyte has a respiratory burst quantitatively comparable to that of the adult cell [21].

The oxidant and antioxidant responses of elderly monocytes were also lower than those observed in monocytes from young adults. Ageing may contribute to the oxidative metabolism dysregulation that affects the elderly. In this regard, controversial information has been reported. Oxidative stress is commonly observed in the elderly and could be involved in age-related diseases [22]. The aging brain undergoes a process of enhanced peroxidative stress, as shown by reports of altered membrane lipids, oxidized proteins, and damaged DNA that could explain age-related neurodegenerative diseases [23]. Experimentally, it has been reported decreased antioxidant enzyme levels with age in murine brain [24]. However, other studies have shown decreased oxidative stress in the elderly. Regarding this, increased activities of antioxidant enzymes (Catalase, SOD) in healthy human neutrophils have been reported to be age –dependent [25]. Decreased plasma membrane fluidity of lymphoid cells and monocytes in advanced age [26], [27] may be one contributor factor to decreased production of NO observed in elderly monocytes, since, it has been reported an association between decreased membrane fluidity in red blood cells of hypertensive patients and low plasma NO-metabolite levels [28]. In addition, monocytes from elderly subjects had a decreased accessory function for PHA-stimulated T cells compare to those obtained from young subjects [13]. Cytokine production and expression of costimulatory T cell proteins (CD80) on monocytes from older adults were lower than those on cells from young individuals [14]. These monocyte alterations could be involved in the course of dengue disease. In this regard, monocytes from neonatal and elderly subjects had decreased production of cytokines after dengue virus interaction compared to young adults, suggesting impairment in the production of cytokines (unpublished data). Since, both immune and oxidative mechanisms could be related, immune alterations could influence oxidative metabolism and vice versa [7]. Thus, the immunosuppressive status in neonatal and elderly monocytes could be involved in decreased oxidant and antioxidant responses after DENV infection.

As an interesting finding, DENV-2 induced higher stimulatory effect on NO production compared to other viral types. This observation could be reflected in patients, since, symptoms, signs, and laboratory findings appear to be different for patients infected with DENV-2 [29].

NO affects virtually every step of the development of inflammation. Low concentrations of NO produced by constitutive nitric oxide synthases (NOS), inhibit adhesion molecule expression, cytokine and chemokine synthesis and leukocyte adhesion and transmigration. Large amounts of NO, generated primarily by the inducible NOS (iNOS) can be toxic and pro-inflammatory [16], [30]. In our study, the amount of NO produced by the different DENV types could be considered toxic, since, increased monocyte content of MDA (lipid peroxidation) was found. High values of NO and MDA were accompanied by increased activities of catalase and SOD and high content of GSH, suggesting an antioxidant response. In this report only NO was studied as an oxidant molecule; however, the induction of oxygen reactive species cannot be discarded, since enzymes such as catalase and SOD were found increased in this study. Of interest, SOD could modulate the oxidant effect of NO, since the interaction of superoxide anion with NO produces peroxynitrites, a high reactive radical [16]. SOD induces the dismutation of superoxide anion diminishing the production of peroxynitrites [31].

The outcome of viral infections depends on viral and host factors. Host cells are thought to respond to viral infection by initiation of apoptotic cell death. There is mounting evidence that dengue virus can trigger the host cell to undergo apoptosis in a cell-dependent manner. During dengue virus infection, cell death is also modulated by the virulence of the infecting strains [32], [33]. In this study, the increased content of NO and MDA in monocytes during infection with all DENV types was accompanied by apoptosis, suggesting that NO was an apoptosis inducer during dengue infection. Dengue viruses generally induce apoptosis in mammalian cells in part, due to oxidative stress [34] and the NO inducer apoptosis role has been reported [35], [36]. The induction of monocyte apoptosis by DENV has previously been shown [36]–[39]. NO can inhibit dengue virus replication by inducing apoptosis, but, other viral inhibitory effects of NO have been reported [40]–[42]. The viral inhibitory effects of NO have been reported in infections by members of different viral families including dengue virus [40], retrovirus [41] and vesicular stomatitis virus [42].

The capacity of all DENV types to induce oxidant/antioxidant effect in monocytes could be relevant during human dengue infection. In this regard, oxidative stress has been reported in dengue associated to severity of disease, thrombocytopenia and increased activity of glutathione peroxidase [4]–[6]. In addition, increased plasma content of NO has also been reported [36], [37], [39], suggesting a role of NO in the oxidative stress during dengue infection.

During this study, both neonatal and elderly monocytes had lower oxidant and antioxidant responses to dengue virus infection than young adult monocytes, suggesting a reduced oxidative response in both ends of life. However, the balance between oxidant/antioxidant effects resulted in lipid peroxidation and apoptosis regardless of monocyte source. Further investigation is required to determine the DENV-induced oxidative/antioxidative responses in monocytes from different sources after macrophage differentiation.

## Supporting Information

Figure S1 Induction of nitric oxide (NO) in neonatal (A), adult (B) and elderly (C) monocytes by dengue virus. Increased production of NO was observed in all virus infected monocyte cultures at days 1 and 3. The highest production was observed in DENV-2 infected cultures.
(TIF)

Figure S2 Malondialdehyde (MDA) content in neonatal (A), adult (B) and elderly (C) monocytes induced by dengue virus. Increased lipid peroxidation was observed in all virus infected monocyte cultures at days 1 and 3. The highest production was observed in DENV-2 or DENV-4 infected cultures.
(TIF)

Figure S3 Catalase activity in neonatal (A), adult (B) and elderly (C) monocytes induced by dengue virus. Increased catalase activity was observed in all virus infected monocyte cultures at days 1 and 3. The highest production was observed in DENV-1 or DENV-4 infected cultures.
(TIF)

Figure S4 Superoxide dismutase activity (SOD) in neonatal (A), adult (B) and elderly (C) monocytes induced by dengue virus. Incremented SOD activity was observed in all virus infected monocyte cultures at days 1 and 3. DENV-1 induced the highest activity of this enzyme.
(TIF)

Figure S5 Reduced glutathione (GSH) content in neonatal (A), adult (B) and elderly (C) monocytes infected by dengue virus. Incremented of GSH content in all virus infected monocyte cultures was observed at days 1 and 3. The highest amount of GSH was observed in cultures infected by DENV-4.
(TIF)

Table S1 (DOCX)

Table S2 (DOCX)

Table S3 (DOCX)

Table S4 (DOCX)

Table S5 (DOCX)

Table S6 (DOCX)
