# Supplementary figures and images for: Differential Oxidative Stress Induced by Dengue Virus in Monocytes from Human Neonates, Adult and Elderly Individuals
Source: PLoS One. 2013 Sep 17;8(9):e73221. doi: 10.1371/journal.pone.0073221 (PMC3775775; doi:10.1371/journal.pone.0073221)

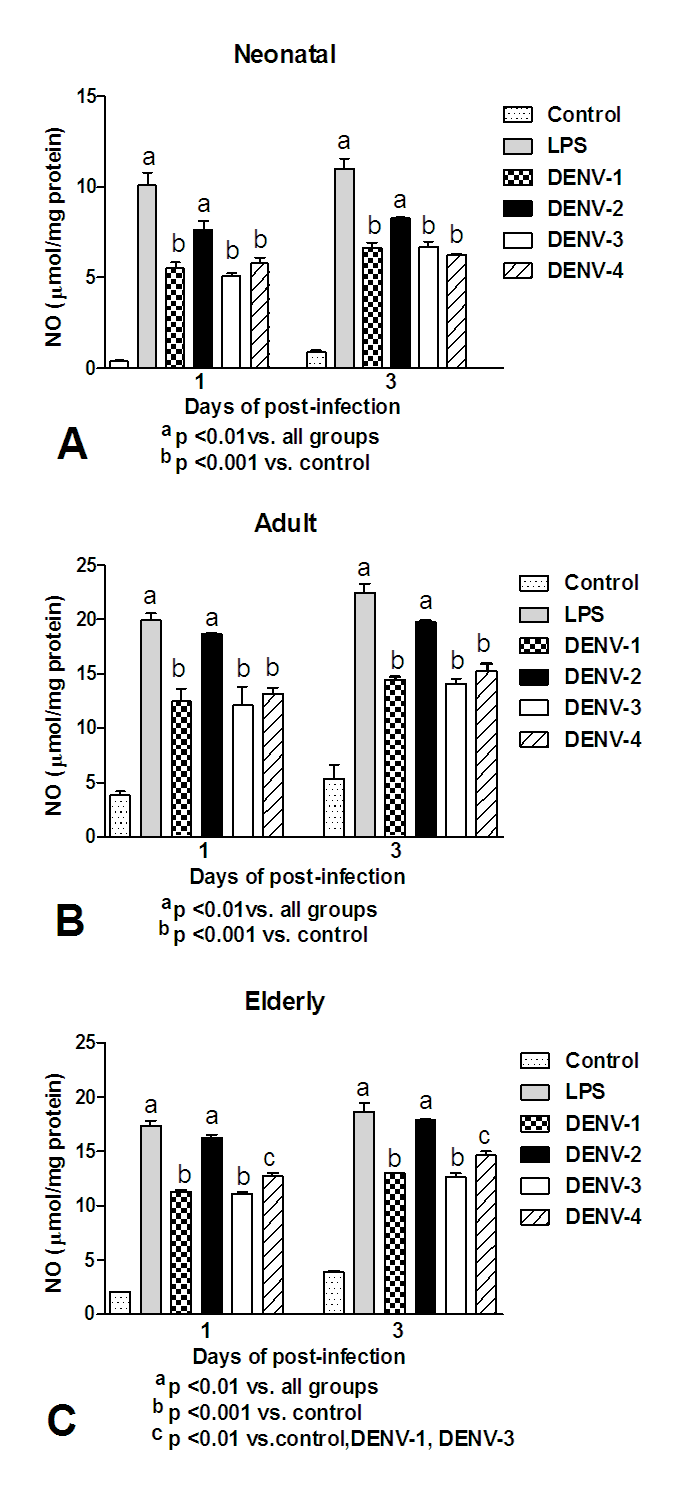

Supplement: Figure S1 — Induction of nitric oxide (NO) in neonatal (A), adult (B) and elderly (C) monocytes by dengue virus. Increased production of NO was observed in all virus infected monocyte cultures at days 1 and 3. The highest production was observed in DENV-2 infected cultures. (TIF) [file pone.0073221.s001.tif]

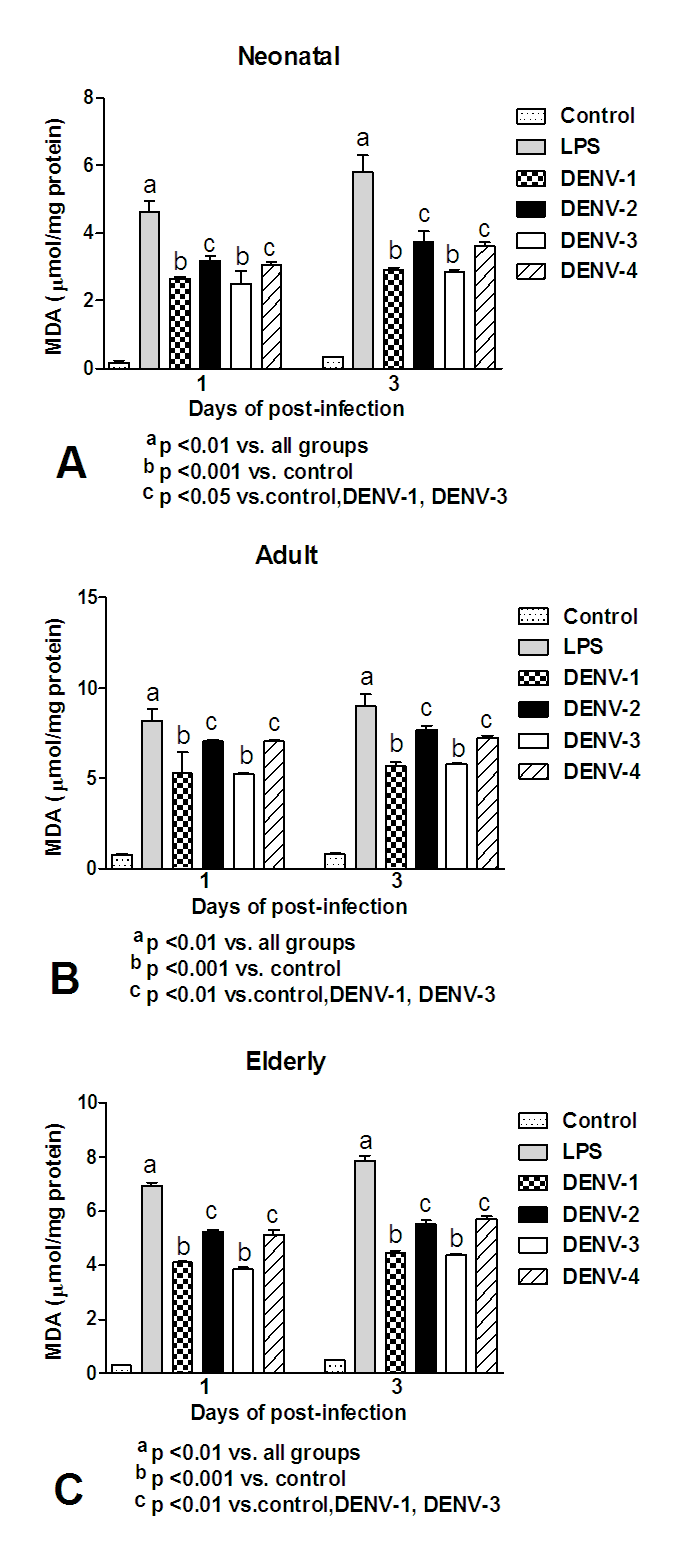

Supplement: Figure S2 — Malondialdehyde (MDA) content in neonatal (A), adult (B) and elderly (C) monocytes induced by dengue virus. Increased lipid peroxidation was observed in all virus infected monocyte cultures at days 1 and 3. The highest production was observed in DENV-2 or DENV-4 infected cultures. (TIF) [file pone.0073221.s002.tif]

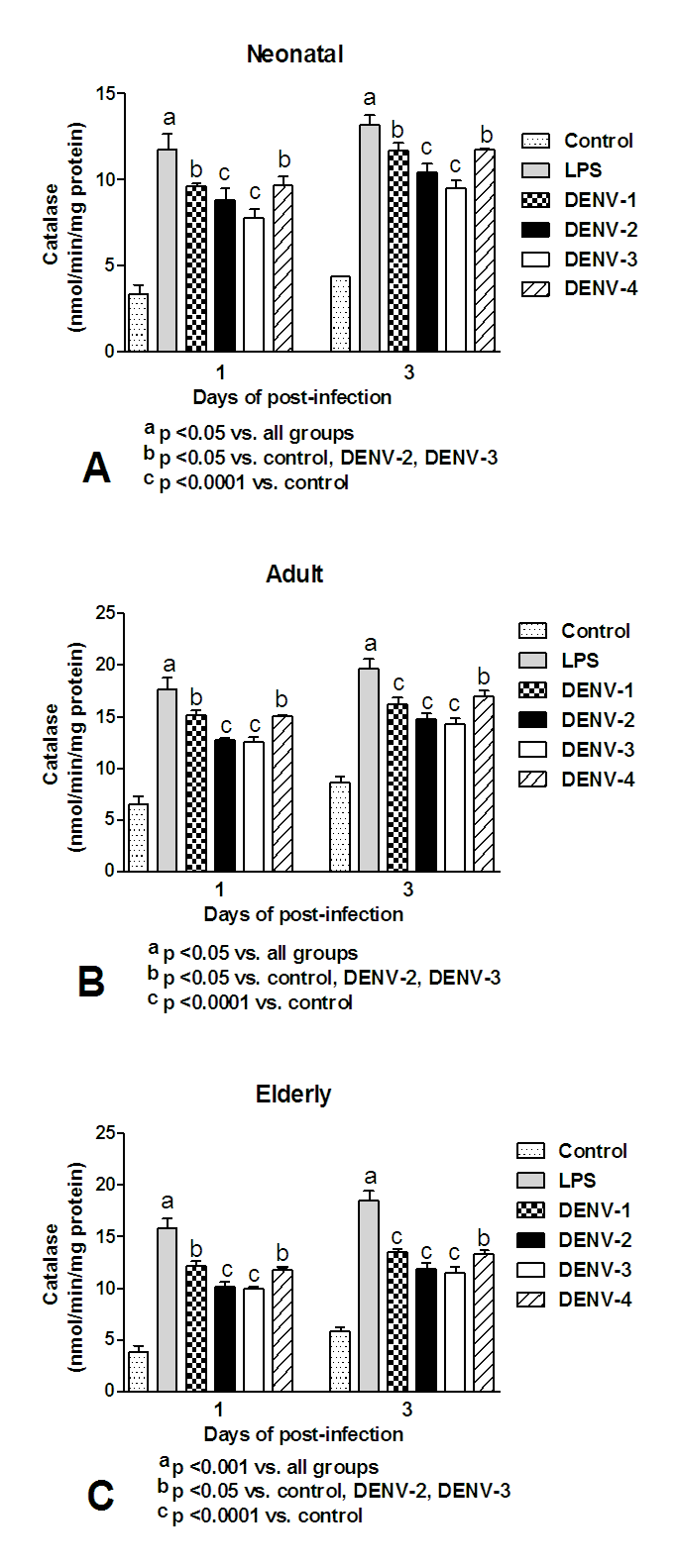

Supplement: Figure S3 — Catalase activity in neonatal (A), adult (B) and elderly (C) monocytes induced by dengue virus. Increased catalase activity was observed in all virus infected monocyte cultures at days 1 and 3. The highest production was observed in DENV-1 or DENV-4 infected cultures. (TIF) [file pone.0073221.s003.tif]

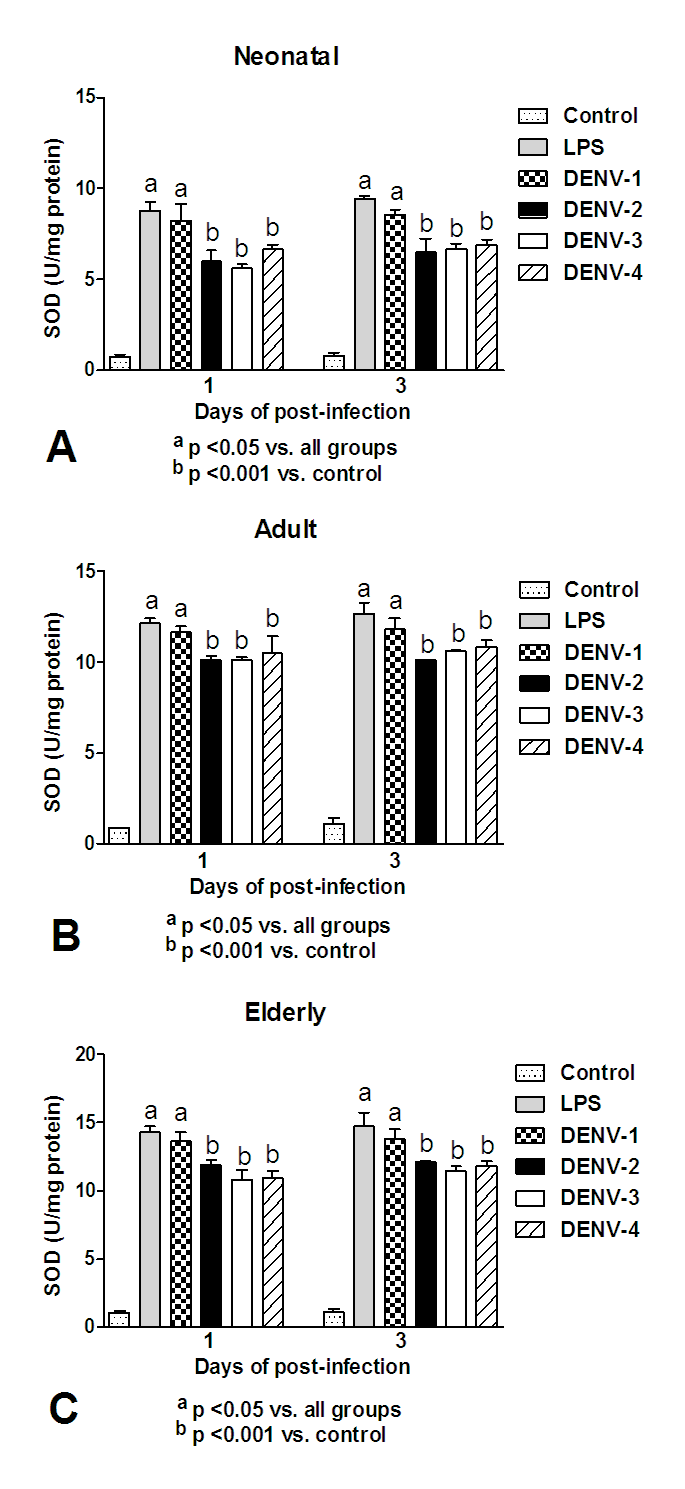

Supplement: Figure S4 — Superoxide dismutase activity (SOD) in neonatal (A), adult (B) and elderly (C) monocytes induced by dengue virus. Incremented SOD activity was observed in all virus infected monocyte cultures at days 1 and 3. DENV-1 induced the highest activity of this enzyme. (TIF) [file pone.0073221.s004.tif]

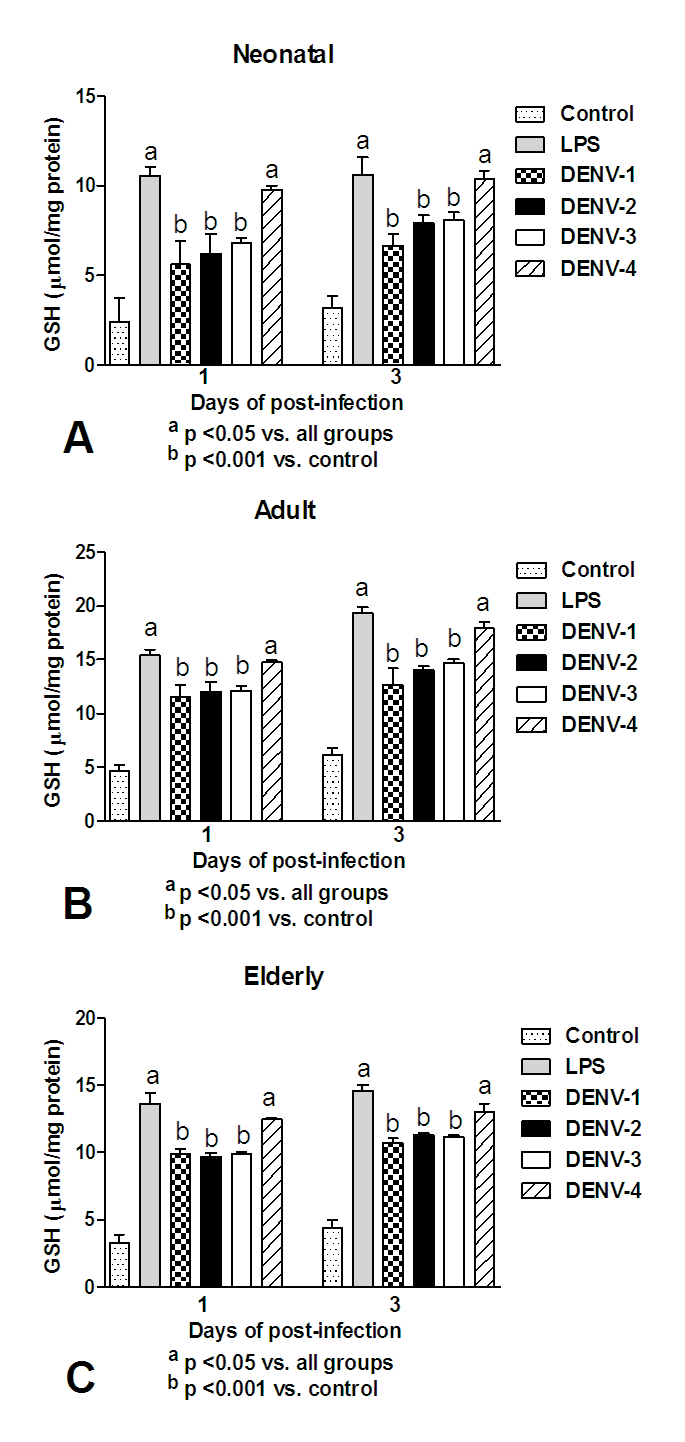

Supplement: Figure S5 — Reduced glutathione (GSH) content in neonatal (A), adult (B) and elderly (C) monocytes infected by dengue virus. Incremented of GSH content in all virus infected monocyte cultures was observed at days 1 and 3. The highest amount of GSH was observed in cultures infected by DENV-4. (TIF) [file pone.0073221.s005.tif]
