# Supplementary material for: Differential Oxidative Stress Induced by Dengue Virus in Monocytes from Human Neonates, Adult and Elderly Individuals
Source: PLoS One. 2013 Sep 17;8(9):e73221. doi: 10.1371/journal.pone.0073221 (PMC3775775; doi:10.1371/journal.pone.0073221)
Supplement: Table S1 — (DOCX) [file pone.0073221.s006.docx]

Table S1. Nitric oxide levels in monocytes from neonates, young and elderly adults infected with dengue virus type -1 to -4.

| Neonatal Elderly Adults | | | | | | |
| --- | --- | --- | --- | --- | --- | --- |
| DENV type | Day 1 p.i. | Day 3 p.i. | Day 1 p.i. | Day 3 p.i. | Day 1 p.i. | Day 3 p.i. |
| DENV-1 | 5.54 ± 0.31 | 6.68 ± 0.26 | 11.23 ± 0.21 | 12.96 ± 0.06 | 12.46 ± 1.14 | 14.44 ± 0.31* |
| DENV-2 | 7.63 ± 0.54 | 11.04 ± 0.56 | 16.30 ± 0.29 | 18.65 ± 0.86 | 18.62 ± 0.09 | 22.42 ± 0.91 |
| DENV-3 | 5.11 ± 0.12 | 6.73 ± 0.24 | 11.06 ± 0.27 | 12.68 ± 0.31 | 12.12 ± 1.68 | 14.11 ± 0.47 |
| DENV-4 | 5.77 ± 0.36 | 6.27 ± 0.05 | 12.71 ± 0.32 | 14.68 ± 0.31 | 13.2 ± 0.55 | 15.27 ± 0.62 |

Data represents mean ± SD. p.i: post infection; * nitric oxide (μM/mg of cellular protein)
