# Supplementary material for: Differential Oxidative Stress Induced by Dengue Virus in Monocytes from Human Neonates, Adult and Elderly Individuals
Source: PLoS One. 2013 Sep 17;8(9):e73221. doi: 10.1371/journal.pone.0073221 (PMC3775775; doi:10.1371/journal.pone.0073221)
Supplement: Table S2 — (DOCX) [file pone.0073221.s007.docx]

Table S2. Malondialdehyde levels in monocytes from neonates, young and elderly adults infected with dengue virus type -1 to -4.

| Neonatal Elderly Adults | | | | | | | | | | | |
| --- | --- | --- | --- | --- | --- | --- | --- | --- | --- | --- | --- |
| DENV type | | Day 1 p.i. | Day 3 p.i. | | Day 1 p.i. | Day 3 p.i. | Day 1 p.i. | | Day 3 p.i. | | |
| DENV-1 | 2.67 ± 0.05 | | 2.92 ± 0.05 | 4.10 ± 0.04 | | 4.46 ± 0.08 | | 5.29 ± 0.15 | | 5.65 ± 0.27* |  |
| DENV-2 | 3.18 ± 0.14 | | 3.78 ± 0.29 | 5.23 ± 0.08 | | 5.53 ± 0.13 | | 7.07 ± 0.06 | | 7.69 ± 0.26 |  |
| DENV-3 | 2.51 ± 0.38 | | 2.86 ± 0.06 | 3.84 ± 0.06 | | 4.39 ± 0.03 | | 5.24 ± 0.07 | | 5.77 ± 0.06 |  |
| DENV-4 | 3.08 ± 0.08 | | 3.63 ± 0.11 | 5.14 ± 0.17 | | 5.70 ± 0.12 | | 7.03 ± 0.12 | | 7.26 ± 0.11 |  |

Data represents mean ± SD. p.i: post infection; * Malondialdehyde (nM/mg of cellular protein)
