# Supplementary material for: Differential Oxidative Stress Induced by Dengue Virus in Monocytes from Human Neonates, Adult and Elderly Individuals
Source: PLoS One. 2013 Sep 17;8(9):e73221. doi: 10.1371/journal.pone.0073221 (PMC3775775; doi:10.1371/journal.pone.0073221)
Supplement: Table S3 — (DOCX) [file pone.0073221.s008.docx]

Table S3. Catalase activity in monocytes from neonates, young and elderly adults infected with dengue virus type -1 to -4.

| Neonatal Elderly Adults | | | | | | | | | |
| --- | --- | --- | --- | --- | --- | --- | --- | --- | --- |
| DENV type | | Day 1 p.i. | Day 3 p.i. | Day 1 p.i. | Day 3 p.i. | Day 1 p.i. | | Day 3 p.i. | |
| DENV-1 | 9.62 ± 0.17 | | 11.71 ± 0.42 | 12.19 ± 0.50 | 13.46 ± 0.34 | | 15.14 ± 0.51 | | 16.21 ± 0.66* |
| DENV-2 | 8.83 ± 0.64 | | 10.43 ± 0.52 | 10.14 ± 0.51 | 11.87 ± 0.62 | | 12.74 ± 0.24 | | 14.72 ± 0.64 |
| DENV-3 | 7.76 ± 0.53 | | 9.47 ± 0.50 | 9.98 ± 0.19 | 11.50 ± 0.62 | | 12.56 ± 0.50 | | 14.29 ± 0.58 |
| DENV-4 | 9.67 ± 0.53 | | 11.74 ± 0.07 | 11.81 ± 0.30 | 13.37 ± 0.34 | | 15.03 ± 0.12 | | 16.98 ± 0.58 |

Data represents mean ± SD. p.i: post infection; * Catalase (nmol/min/mg protein)
