# Supplementary material for: Differential Oxidative Stress Induced by Dengue Virus in Monocytes from Human Neonates, Adult and Elderly Individuals
Source: PLoS One. 2013 Sep 17;8(9):e73221. doi: 10.1371/journal.pone.0073221 (PMC3775775; doi:10.1371/journal.pone.0073221)
Supplement: Table S4 — (DOCX) [file pone.0073221.s009.docx]

Table S4. Superoxide dismutase levels in monocytes from neonates, young and elderly adults infected with dengue virus type -1 to -4.

| Neonatal Elderly Adults | | | | | | | | | |
| --- | --- | --- | --- | --- | --- | --- | --- | --- | --- |
| DENV type | | Day 1 p.i. | Day 3 p.i. | Day 1 p.i. | Day 3 p.i. | Day 1 p.i. | | Day 3 p.i. | |
| DENV-1 | 8.23 ± 0.92 | | 8.54 ± 0.30 | 13.66 ± 0.63 | 13.78 ± 0.73 | | 11.63 ± 0.37 | | 11.79 ± 0.64* |
| DENV-2 | 5.97 ± 0.64 | | 6.49 ± 0.76 | 11.87 ± 0.35 | 12.09 ± 0.11 | | 10.11 ± 0.23 | | 10.12 ± 0.02 |
| DENV-3 | 5.62 ± 0.24 | | 6.63 ± 0.32 | 10.82 ± 0.69 | 11.41 ± 0.39 | | 10.11 ± 0.18 | | 10.62 ± 0.06 |
| DENV-4 | 6.64 ± 0.27 | | 6.84 ± 0.36 | 10.94 ± 0.59 | 11.83 ± 0.39 | | 10.49 ± 0.97 | | 10.82 ± 0.40 |

Data represents mean ± SD. p.i: post infection; * Superoxide dismutase (U/mg protein)
