# Supplementary material for: Differential Oxidative Stress Induced by Dengue Virus in Monocytes from Human Neonates, Adult and Elderly Individuals
Source: PLoS One. 2013 Sep 17;8(9):e73221. doi: 10.1371/journal.pone.0073221 (PMC3775775; doi:10.1371/journal.pone.0073221)
Supplement: Table S5 — (DOCX) [file pone.0073221.s010.docx]

Table S5. Reduced glutathione levels in monocytes from neonates, young and elderly adults infected with dengue virus type -1 to -4.

| Neonatal Elderly Adults | | | | | | | | | |
| --- | --- | --- | --- | --- | --- | --- | --- | --- | --- |
| DENV type | | Day 1 p.i. | Day 3 p.i. | Day 1 p.i. | Day 3 p.i. | Day 1 p.i. | | Day 3 p.i. | |
| DENV-1 | 5.59 ± 1.31 | | 6.63 ± 0.68 | 9.92 ± 0.32 | 10.71 ± 0.34 | | 11.53 ± 1.10 | | 12.67 ± 1.53* |
| DENV-2 | 6.22 ± 1.10 | | 7.92 ± 0.44 | 9.69 ± 0.30 | 11.29 ± 0.12 | | 12.04 ± 0.84 | | 14.02 ± 0.38 |
| DENV-3 | 6.81 ± 0.26 | | 8.06 ± 0.32 | 9.92 ± 0.12 | 11.11 ± 0.17 | | 12.11 ± 0.48 | | 14.64 ± 0.41 |
| DENV-4 | 9.76 ± 0.24 | | 10.39 ± 0.44 | 12.47 ± 0.08 | 13.07 ± 0.52 | | 14.74 ± 0.20 | | 17.97 ± 0.54 |

Data represents mean ± SD. p.i: post infection; * Reduced glutathione (nM/mg of cellular protein)
