# Supplementary material for: Differential Oxidative Stress Induced by Dengue Virus in Monocytes from Human Neonates, Adult and Elderly Individuals
Source: PLoS One. 2013 Sep 17;8(9):e73221. doi: 10.1371/journal.pone.0073221 (PMC3775775; doi:10.1371/journal.pone.0073221)
Supplement: Table S6 — (DOCX) [file pone.0073221.s011.docx]

Table S6. Percentage of apoptotic monocytes from neonates, young and elderly adults infected with dengue virus type -1 to -4.

| Neonatal Elderly Adults | | | | | | | | | | | |  |
| --- | --- | --- | --- | --- | --- | --- | --- | --- | --- | --- | --- | --- |
| Stimuli | | Day 1 p.i. | Day 3 p.i. | Day 1 p.i. | | Day 3 p.i. | | Day 1 p.i. | | Day 3 p.i. | |  |
| Control | 8.98 ± 2.58 | | 9.85 ± 3.16 | | 6.98 ± 1.32 | | 12.44 ± 1.86 | | 7.08 ± 1.86 | | 11.0 ± 2.86* | |
| LPS | 55.36 ± 3.55 | | 69.87 ± 2.37 | | 52.63 ± 2.85 | | 68.99 ± 4.90 | | 50.36 ± 2.56 | | 67.85 ± 5.64 | |
| DENV-1 | 57.65 ± 3.52 | | 76.84 ± 5.32 | | 50.36 ± 3.21 | | 70.87 ± 4.97 | | 52.12 ± 3.55 | | 73.85 ± 6.85 | |
| DENV-2 | 58.36 ± 1.57 | | 75.54 ± 3.86 | | 53.37 ± 3.38 | | 69.84 ± 5.90 | | 51.26 ± 4.86 | | 70.23 ± 7.99 | |
| DENV-3 | 57.12 ± 5.33 | | 76.77 ± 3.84 | | 50.87 ± 3.97 | | 72.62 ± 6.14 | | 51.85 ± 2.54 | | 71.65 ± 4.78 | |
| DENV-4 | 52.14 ± 4.80 | | 75.60 ± 3.30 | | 51.97 ± 4.91 | | 73.36 ± 7.32 | | 52.85 ± 5.82 | | 72.74 ± 5.70 | |

Data represents mean ± SD. p.i: post infection; LPS: lipopolysaccharide * Apoptosis percentage of TUNEL positive cells
